# Antiapolipoprotein A-1 Autoantibody Positivity Is Associated with Threatened Abortion

**DOI:** 10.1155/2020/9309121

**Published:** 2020-03-07

**Authors:** Alessandra Vecchié, Aldo Bonaventura, Federico Carbone, Davide Maggi, Antonella Ferraiolo, Beatrice Carloni, Gabriella Andraghetti, Laura Affinito Bonabello, Luca Liberale, Vanessa Fetaud, Sabrina Pagano, Franco Dallegri, Renzo Cordera, Fabrizio Montecucco, Nicolas Vuilleumier

**Affiliations:** ^1^First Clinic of Internal Medicine, Department of Internal Medicine, University of Genoa, 6 Viale Benedetto XV, 16132 Genoa, Italy; ^2^Virginia Commonwealth University, Pauley Heart Center, Division of Cardiology, Department of Internal Medicine, 1200 East Marshall Street, 23298 Richmond, Virginia, USA; ^3^IRCCS Ospedale Policlinico San Martino Genova-Italian Cardiovascular Network, 10 Largo Benzi, 16132 Genoa, Italy; ^4^Diabetology Unit, Department of Internal Medicine, University of Genoa, 6 Viale Benedetto XV, 16132 Genoa, Italy; ^5^Department of Obstetrics and Gynecology, IRCCS Ospedale Policlinico San Martino Genova, 10 Largo Benzi, 16132 Genoa, Italy; ^6^Department of Internal Medicine, University of Genoa, 6 Viale Benedetto XV, 16132 Genoa, Italy; ^7^Center for Molecular Cardiology, University of Zurich, 12 Wagistrasse, 8952 Schlieren, Switzerland; ^8^Division of Laboratory Medicine, Department of Genetics and Laboratory Medicine, Geneva University Hospitals, 4 Rue Gabrielle-Perret-Gentil, 1205 Geneva, Switzerland; ^9^Division of Laboratory Medicine, Department of Medical Specialties, Geneva Faculty of Medicine, Switzerland; ^10^First Clinic of Internal Medicine, Department of Internal Medicine and Center of Excellence for Biomedical Research (CEBR), University of Genoa, 6 Viale Benedetto XV, 16132 Genoa, Italy

## Abstract

**Background:**

Autoantibodies against apolipoprotein A-1 (anti-ApoA-1 IgG) were demonstrated to be associated with cardiovascular outcomes in several inflammatory diseases. As balanced inflammation is critical for uncomplicated pregnancy, we aimed to investigate the prevalence of anti-ApoA-1 IgG and anti-c-terminal ApoA-1 autoantibodies (Ac-terAA1 IgG) in a cohort of pregnant women and their potential relationship with threatened abortion (TA).

**Methods:**

Between 2012 and 2014, 371 consecutive outpatient pregnant women were included in this study and followed until delivery. Anti-ApoA-1 and anti-Ac-terAA1 IgG were measured by ELISA technique on serum samples collected between the 24^th^ and 26^th^ week of pregnancy. Associations with TA were tested using linear regression analysis and C-statistics.

**Results:**

Median age was 34 with a prevalence of the Caucasian ethnicity (90.5%). TA occurred in 10 women (2.7%). C-statistics indicated that anti-ApoA-1 and anti-Ac-terAA1 IgG levels upon study inclusion were predictive of TA (0.73, 95% confidence interval [CI] 0.69-0.78, *p* < 0.001 and 0.76, 95% CI 0.71-0.80, *p* < 0.001 and 0.76, 95% CI 0.71-0.80, *p* < 0.001 and 0.76, 95% CI 0.71-0.80, *p* < 0.001 and 0.76, 95% CI 0.71-0.80,

**Conclusion:**

Anti-ApoA-1 and anti-Ac-terAA1 IgG are independently associated with TA during pregnancy with an appealing NPV. The causal biological mechanisms underlying this association as well as the possible clinical relevance of these findings require further investigations.

## 1. Introduction

Several autoimmune diseases (i.e., anti-phospholipid [APL] syndrome [APS] and systemic lupus erythematosus [SLE]) characterized by the production of APL autoantibodies are known to be associated with recurrent vascular thrombosis and/or obstetrical complications, including abortion, where APL autoantibodies are believed to play a causal role [1, 2]. Furthermore, in approximately 1-6% of healthy women, the presence of high levels of APL autoantibodies can be detected and can potentially affect physiological pregnancy [1, 2]. As well, we recently found that even moderately high levels of C-reactive protein (CRP), a classical biomarker of systemic inflammation, can help in recognizing pregnant women at increased risk for adverse outcomes [3], thus highlighting the pivotal effect that inflammation may have during pregnancy.

In APS and SLE, APL autoantibodies were shown to be associated with antibodies against apolipoprotein A-1 (anti-ApoA-1 IgG), the major fraction of high-density lipoproteins, and such association may be explained by a certain degree of cross-reactivity between APL and anti-ApoA-1 antibodies [3, 4]. The seropositivity for the latter antibodies is fairly prevalent in the general population (approximately 20%) and represents an independent predictor of death and acute cardiovascular events associated with vascular inflammation [4, 5]. Furthermore, experimental studies indicate that these antibodies can promote atherogenesis and atherothrombosis by acting on Toll-like receptors 2 and 4 [6–8]. Because of their possible cross-reactivity with APL antibodies and their proinflammatory effects, we hypothesized that anti-ApoA-1 IgG could jeopardize the balanced inflammatory process [4] involved in physiological pregnancy [9], promoting a proinflammatory systemic environment associated with the development of maternal complications and neonatal diseases [10]. Since the polyclonal anti-ApoA-1 IgG response in humans was shown to be oriented against the last *α*-helix of c-terminus part of native apoA-1 (amino acids: 220-242) [4, 11, 12], we aimed to determine the prevalence of both anti-ApoA-1 and anti-c-terminus apoA-1 (anti-Ac-terAA1) IgG and their possible relationship with threatened abortion (TA) in a general population of pregnant women.

## 2. Materials and Methods

### 2.1. Study Population and Clinical Assessment

As previously described [3], 380 consecutive outpatient pregnant women between the 24^th^ and 26^th^ gestational week (gestational age 23 weeks + 0 days–25 weeks + 6 days) aged 18 or older attending the Diabetology Unit of IRCCS Ospedale Policlinico San Martino (Genoa, Italy) were enrolled from October 2012 to November 2014. No other specific inclusion criteria than a pregnant status were established, while exclusion criteria consisted in clinical suspicion of an active infection or concurrent treatment with corticosteroids (*n* = 9), thus leaving 371 patients eligible for analyses. Methods for collection of serum samples as well as maternal adverse outcomes other than prevalent TA were already described elsewhere [3]. The Ethics Committee of the IRCCS Ospedale Policlinico San Martino (Genoa, Italy) approved this study, which was performed in accordance with the guidelines of the Declaration of Helsinki.

### 2.2. Study Endpoint Definition

The primary endpoint of our study was to evaluate the association of anti-ApoA-1 IgG and anti-Ac-terAA1 IgG with TA in this cohort of pregnant women. The secondary endpoint was to determine the prevalence of anti-ApoA-1 IgG and anti-Ac-terAA1 IgG in the enrolled subjects.

TA is an adverse maternal outcome that is defined based on the occurrence of vaginal bleeding with or without abdominal pain in the first 20 weeks of pregnancy, but diagnostic criteria for spontaneous abortion are not met [13].

### 2.3. Detection of Anti-ApoA-1 and Anti-Ac-terAA1 IgG and of C-Reactive Protein

Anti-ApoA-1 IgG serum levels were measured as previously described [6, 14]. In sum, MaxiSorp plates (Nunc) were coated with purified, human-derived delipidated apoA-1 or chemically engineered c-terAA1 [12] (20 *μ*g/mL; 50 *μ*L/well for both protein) for 1 hour at 37°C. Following three washes with phosphate buffered saline (PBS)/2% bovine serum albumin (BSA; 100 *μ*L/well), all wells were blocked for 1 hour with 2% BSA at 37°C. Samples were diluted 1 : 50 in PBS/2% BSA and incubated for 60 minutes. Other patient samples at the same dilution were also added to an uncoated well to assess individual nonspecific binding. After six further washes, 50 *μ*L/well of signal antibody (alkaline phosphatase-conjugated antihuman IgG; Sigma-Aldrich) diluted 1 : 1,000 in PBS/2% BSA solution was incubated for 1 hour at 37°C. After six more washes (150 *μ*L/well) with PBS/2% BSA solution, the phosphatase substrate p-nitrophenyl phosphate disodium (50 *μ*L/well; Sigma-Aldrich) dissolved in diethanolamine buffer (pH 9.8) was added. Each sample was tested in duplicate, and absorbance, determined as the optical density (OD) at 405 nm, was assessed after 20 minutes of incubation at 37°C (VersaMax, Molecular Devices). The corresponding nonspecific binding value was subtracted from the mean absorbance value for each sample. For anti-ApoA-1 IgG, the positivity cutoff was set at an OD value of 0.6 and 37% of the positive control value as described and validated in previous studies [6, 14]. OD values ranged from 0 to 1.78, and corresponding index values were between 0 and 99.2%. For anti-Ac-terAA1 IgG, OD values ranged between 0.21 and 1.45, and the index values ranged between 13 and 91%. By analogy, anti-Ac-terAA1 IgG seropositivity cutoff was set at the 97.5 centile of the distribution obtained on 160 healthy blood donors [15], which corresponded to an OD value above 0.5 and an index above 37% of the positive control. For both assays, at the seropositivity cutoffs, the interassay coefficients of variation were below 9% and the intra-assay CV below 5%.

Serum levels of CRP were measured by colorimetric enzyme-linked immunosorbent assay (ELISA) following the manufacturer's instructions (R&D Systems, Minneapolis, MN). The limit of detection was 15.625 pg/mL. Mean intra- and interassay coefficients of variation were <8%.

### 2.4. Statistical Analysis

Categorical data were presented as relative and absolute frequencies and compared with chi-square or Fisher's exact test, as appropriate, while continuous variables were shown as median and interquartile range (IQR) and their comparison was performed by nonparametric Mann–Whitney *U* test. The correlation between anti-ApoA-1 and anti-Ac-terAA1 IgG was calculated by Spearman's rank correlation test. The association between anti-ApoA-1 and anti-Ac-terAA1 IgG and TA was calculated by a linear regression analysis and expressed with 95% confidence interval (95% CI). The prognostic ability of the two autoantibodies towards the prediction of TA was measured by C-statistics, whose value was given with corresponding 95% CI. For the cutoffs of both anti-ApoA-1 and anti-Ac-terAA1 IgG, the corresponding sensitivity, specificity, and positive and negative predictive values (PPV and NPV, respectively) were provided. Analyses were performed using IBM Statistical Package for Social Science (SPSS) for Windows, Version 25.0 (IBM Co., Armonk, NY) and MedCalc 12.5 (MedCalc Software, Ostend, Belgium).

## 3. Results

### 3.1. Patients' Characteristics

Patients' clinical characteristics are reported in Table 1. Median age was 34 (31-37), and there was a substantial prevalence of the Caucasian race (90.5%). Median pre-pregnancy body mass index was 21.19 kg/m^2^ (19.80-23.57), while median CRP was 3.35 *μ*g/mL (1.62-8.28).

Most frequent comorbidities were gestational diabetes (11.9%), autoimmune diseases (6.7%), and thyroid disease (6.5%), while coagulopathies (including platelet disorders, such as gestational thrombocytopenia or idiopathic thrombocytopenic purpura) and polycystic ovary syndrome accounted for 1.9%. Among autoimmune diseases, Hashimoto thyroiditis was the most frequent one (4.9%) followed by SLE (0.5%), while Basedow's disease, Behçet syndrome, APS, chronic gastritis, and multiple sclerosis affected one woman each. Ten women (2.7%) experienced TA (Table 1). Available adverse maternal outcomes (other than TA) are summarized in Supplementary [Supplementary-material supplementary-material-1].

According to previously validated and predetermined cutoffs [6, 14, 15], 31 subjects (8.4%) were found positive for anti-ApoA-1 IgG and 82 (22.1%) for anti-Ac-terAA1 IgG. Despite this seropositivity prevalence difference, a positive correlation between anti-ApoA-1 and anti-Ac-terAA1 IgG was established (Supplementary [Supplementary-material supplementary-material-1]). No correlation, however, was found for anti-ApoA-1 and anti-Ac-terAA1 IgG with systemic inflammation in terms of CRP (*r* = −0.094, *p* = 0.070 and *r* = −0.027, *p* = 0.600, respectively).

When comparing women based on positivity/negativity for anti-ApoA-1 and anti-Ac-terAA1 IgG (Tables 2 and 3), no difference was found for comorbidities (with special reference to autoimmune diseases), therapies, and overall maternal adverse outcomes (Supplementary Tables [Supplementary-material supplementary-material-1]). However, TA occurrence was significantly increased among positive individuals as compared with negative ones for both autoantibodies (Tables 2 and 3). With regard to ethnicity, anti-Ac-terAA1 IgG positive women were more frequently of ethnicity other than Caucasian (Table 3), but such association was not found for anti-ApoA-1 IgG (Table 2).

### 3.2. Anti-ApoA-1 IgG, Anti-Ac-terAA1 IgG, and Threatened Abortion Occurrence

Linear regression analyses showed a significant association between TA and anti-ApoA-1 IgG levels in the univariate model (*β* = 0.162, 95% CI 0.071-0.253, *p* < 0.001, Table 4). This result was confirmed also in the multivariate model when considering age and the presence of autoimmune diseases as potential confounders (*β* = 0.181, 95% CI 0.072-0.255, *p* < 0.001, Table 4). Similarly, anti-Ac-terAA1 IgG levels were associated with TA in the multivariate model (*β* = 0.179, 95% CI 0.067-0.240, *p* = 0.001, Table 4).

**Figure 1:**
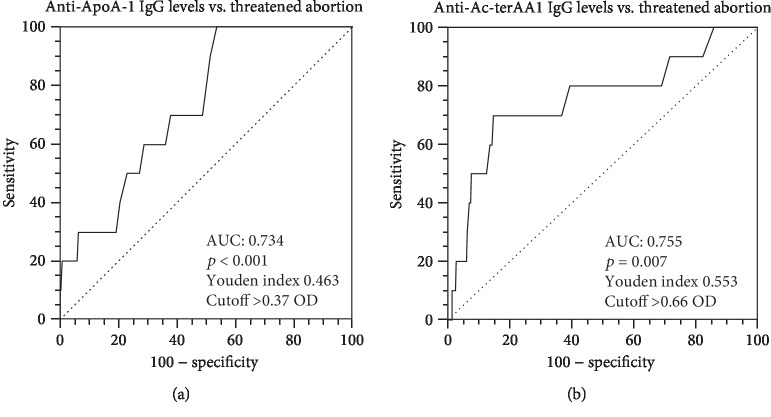
C-statistics analysis for anti-apoA-1 IgG (a) and anti-Ac-terAA1 IgG (b) levels toward threatened abortion.

These results were corroborated by C-statistics analyses, indicating that both anti-ApoA-1 and anti-Ac-terAA1 IgG serum levels during pregnancy displayed significant prognostic accuracy for TA (0.73, 95% CI 0.69-0.78, *p* < 0.001 and 0.76, 95% CI 0.71-0.80, *p* = 0.007, Figures 1(a) and 1(b), respectively). At the predetermined cutoff of anti-ApoA-1 IgG set at OD >0.4 and an index >37%, the test had a sensitivity of 100% and a specificity of 46.3% with a PPV of 4.9% and an NPV of 100%. According to C-statistics optimal cutoff set at OD >0.7, anti-Ac-terAA1 IgG showed a sensitivity and a specificity of 70 and 85.3%, respectively, with a PPV of 11.7% and an NPV of 99.0%.

## 4. Discussion

The main novelty of our study is that both anti-ApoA-1 and anti-Ac-terAA1 IgG are associated with TA in a general cohort of pregnant women. At the best of our knowledge, the role of these autoantibodies was not investigated before in gestation and represents a new field of research linking autoantibodies and pregnancy complications.

TA represents a serious pregnancy complication and is associated with an increased risk for adverse outcomes [16]. Abortion frequently occurs when fetus is affected by chromosomal abnormalities, but it may be also favored by several maternal factors, such as extreme weight (high or low) [17], older age, unhealthy lifestyle habits [18], and chronic illnesses, particularly autoimmune diseases (e.g., SLE) [19]. Both systemic and local inflammatory processes are believed to have a central role in abortion pathophysiology [20]. Since anti-ApoA-1 IgG were shown to promote a dose-dependent production of proinflammatory cytokines (interleukin [IL]-6, IL-8, and TNF-*α*, and matrix metalloproteinase-9) [6], an inflammatory link might be suspected between higher levels of these antibodies and TA. Therefore, as these cytokines stimulate T-helper 2 polarization [20] required for a successful pregnancy [21] and no correlation with CRP was found in the present study, other factors should be considered.

APL autoantibodies are a well-known risk factor for the development of obstetric and maternal complications [22]. Particularly, obstetric APS is the most frequent acquired risk factor for recurrent pregnancy loss [23]. In a recent study, APL positivity was associated with pregnancy adverse outcomes, independently of a confirmed diagnosis of APS and from treatment with aspirin or heparin [24]. According to recent studies, APL positivity may be more common than previously thought in the general population [22]. Further investigations, therefore, are warranted to explore the association of each antibody with pregnancy adverse outcomes.

Interestingly, women positive for anti-Ac-terAA1 IgG were more frequently of ethnicity other than Caucasian compared with negative ones, and such association was not found for anti-ApoA-1 IgG. By considering the results in our cohort, we may hypothesize that some ethnic groups show a predisposition to produce anti-Ac-terAA1 rather than anti-ApoA-1 antibodies. This hypothesis could be explained by the fact that the human polyclonal response is oriented against the c-ter part of apoA-1 [11, 12], as herein demonstrated by the larger seropositivity for anti-Ac-terAA1 IgG compared with anti-ApoA-1 IgG. Whether such difference may have clinical implication remains still elusive, and other population studies are needed to confirm these preliminary results.

We have to acknowledge some limitations in this observational study. First of all, the number of TAs is low in this cohort, so a further evaluation in a larger population is needed to confirm our results. Secondly, in this retrospective analysis, only women with full-term pregnancy were considered, while we do not have any data on women experiencing abortion to make a comparison and evaluate a possible role for abortion. Finally, data on lifestyle habits were not available, which might partially influence the analysis of pregnancy outcomes.

## 5. Conclusion

In conclusion, high levels of anti-ApoA-1 IgG and anti-Ac-terAA1 during pregnancy are associated with TA confirming previous data on inflammation, when impaired, as a possible risk factor for abortion. Also, a different prevalence of anti-Ac-terAA1 IgG positivity emerged in our study according to ethnicity, which might represent a clinically relevant issue to be further investigated.

Although we found that anti-ApoA-1 IgG, a pro-inflammatory factor already validated in larger cohorts of patients suffering from cardiovascular disease, might negatively impact on gestation, larger studies are warranted to confirm these results.

## Figures and Tables

**Table 1:** Baseline characteristics of the overall cohort.

	*n* = 371
Demographics/biomarkers	
Age at enrollment (years)	34 (31-37)
Ethnicity	
Caucasian, *n* (%)	333 (90.5)
Latin American, *n* (%)	20 (5.4)
African, *n* (%)	6 (1.6)
Indian, *n* (%)	9 (2.4)
BMI (kg/m^2^)	21 (20-24)
Weight gain at delivery (kg)	12 (10-15)
CRP (*μ*g/mL)	3.35 (1.62-8.28)
Comorbidities	
Thyroid disease, *n* (%)	24 (6.5)
Hypertension, *n* (%)	5 (1.3)
PCOS, *n* (%)	7 (1.9)
Coagulopathies, *n* (%)	7 (1.9)
Autoimmune diseases, *n* (%)	25 (6.7)
Hashimoto thyroiditis, *n* (%)	18 (4.9)
Basedow's disease, *n* (%)	1 (0.3)
SLE, *n* (%)	2 (0.5)
Behçet syndrome, *n* (%)	1 (0.3)
APS, *n* (%)	1 (0.3)
Chronic gastritis, *n* (%)	1 (0.3)
Multiple sclerosis, *n* (%)	1 (0.3)
GDM, *n* (%)	44 (11.9)
Therapy	
Thyroid hormone replacement, *n* (%)	21 (5.7)
Aspirin, *n* (%)	13 (3.5)
Primary outcome	
Threatened abortion, *n* (%)	10 (2.7)
Anti-ApoA-1 IgG	
Anti-ApoA-1 IgG positivity, *n* (%)	31 (8.4)
Anti-ApoA-1, OD	0.39 (0.30-0.51)
Anti-Ac-terAA1 IgG positivity, *n* (%)	82 (22.1)
Anti-Ac-terAA1 IgG, OD	0.45 (0.36-0.58)

Data are presented as a number and percentage of all cases or as median and interquartile range (IQR). Ac-terAA1 IgG: anti-c-terminus apoA-1 autoantibody; Anti-apoA-1 IgG: apoliprotein A-1 autoantibody; APS: antiphospholipid syndrome; BMI: body mass index; CRP: C-reactive protein; GDM: gestational diabetes mellitus; OD: optical density; PCOS: polycystic ovary syndrome; SLE: systemic lupus erythematosus.

**Table 2:** Baseline characteristics of the overall cohort according to positivity/negativity for anti-ApoA-1 IgG autoantibodies.

	Anti-ApoA-1 IgG negative (*n* = 340)	Anti-ApoA-1 IgG positive (*n* = 31)	*p*
Demographics/biomarkers			
Age at enrollment (years)	34 (31-37)	32 (29-36)	0.082
Ethnicity			0.355
Caucasian, *n* (%)	308 (90.9)	25 (86.2)	0.503
Latin American, *n* (%)	18 (5.3)	2 (6.9)	0.665
African, *n* (%)	6 (1.8)	0 (0)	1.000
Indian, *n* (%)	7 (2.0)	2 (6.9)	0.153
BMI (kg/m^2^)	21 (20-24)	22 (20-26)	0.303
Weight gain at delivery (kg)	12 (10-15)	13 (9-18)	0.930
CRP (*μ*g/mL)	3.36 (1.57-8.65)	2.72 (1.84-6.93)	0.715
Comorbidities			
Thyroid disease, *n* (%)	22 (6.5)	2 (6.5)	1.000
Hypertension, *n* (%)	5 (1.5)	0 (0)	1.000
PCOS, *n* (%)	7 (2.1)	0 (0)	1.000
Coagulopathies, *n* (%)	7 (2.1)	0 (0)	1.000
Autoimmune diseases, *n* (%)	23 (6.8)	2 (6.5)	1.000
Hashimoto thyroiditis, *n* (%)	16 (4.7)	2 (6.5)	0.655
Basedow's diseases, *n* (%)	1 (0.3)	0 (0)	1.000
SLE, *n* (%)	2 (0.6)	0 (0)	1.000
Behçet syndrome, *n* (%)	1 (0.3)	0 (0)	1.000
APS, *n* (%)	1 (0.3)	0 (0)	1.000
Chronic gastritis, *n* (%)	1 (0.3)	0 (0)	1.000
Multiple sclerosis, *n* (%)	1 (0.3)	0 (0)	1.000
GDM, *n* (%)	39 (11.5)	5 (16.1)	0.450
Therapy			
Thyroid hormone replacement, *n* (%)	19 (5.6)	2 (6.5)	0.691
Aspirin, *n* (%)	13 (3.8)	0 (0)	1.000
Pregnancy and delivery characteristics			
Threatened abortion, *n* (%)	7 (2.1)	3 (9.7)	**0.042**

Data are presented as a number and percentage (%) of all cases or as median and interquartile range (IQR). *p* values have been calculated according to chi-square or Fisher's exact test or Mann–Whitney test, as appropriate, and referred to as comparisons between study groups. Statistically significant correlations have been highlighted in bold character. Anti-ApoA-1 IgG: apoliprotein A-1 autoantibody; APS: antiphospholipid syndrome; BMI: body mass index; CRP: C-reactive protein; GDM: gestational diabetes mellitus; PCOS: polycystic ovary syndrome; SLE: systemic lupus erythematosus.

**Table 3:** Clinical characteristics of the cohort according to positivity/negativity for anti-c-terminus apoA-1 (Ac-terAA1) IgG autoantibodies.

	Anti-Ac-terAA1 IgG negative (*n* = 289)	Ac-terAA1 IgG positive (*n* = 82)	*p*
Demographics/biomarkers			
Age at enrollment (years)	34 (31-37)	33 (30-36)	0.256
Ethnicity			**0.005**
Caucasian, *n* (%)	267 (92.7)	66 (82.5)	**0.009**
Latin American, *n* (%)	14 (4.9)	6 (7.5)	0.402
African, *n* (%)	4 (1.4)	2 (2.5)	0.615
Indian, *n* (%)	3 (1.0)	6 (7.5)	**0.004**
BMI (kg/m^2^)	21 (20-24)	21 (20-24)	0.691
Weight gain at delivery (kg)	12 (11-16)	11 (9-15)	0.209
CRP (*μ*g/mL)	3.32 (1.52-8.73)	3.78 (1.85-7.29)	0.936
Comorbidities			
Thyroid disease, *n* (%)	17 (5.9)	7 (8.5)	0.444
Hypertension, *n* (%)	4 (1.4)	1 (1.2)	1.000
PCOS, *n* (%)	6 (2.1)	1 (1.2)	1.000
Coagulopathies, *n* (%)	7 (2.4)	0 (0)	0.356
Autoimmune diseases, *n* (%)	18 (6.2)	7 (8.5)	0.458
Hashimoto thyroiditis, *n* (%)	12 (4.2)	6 (7.3)	0.248
Basedow's disease, *n* (%)	1 (0.3)	0 (0)	1.000
SLE, *n* (%)	1 (0.3)	1 (1.2)	0.394
Behçet syndrome, *n* (%)	1 (0.3)	0 (0)	1.000
APS, *n* (%)	1 (0.3)	0 (0)	1.000
Chronic gastritis, *n* (%)	1 (0.3)	0 (0)	1.000
Multiple sclerosis, *n* (%)	1 (0.3)	0 (0)	1.000
GDM	35 (12.2)	9 (11)	0.764
Therapy			
Thyroid hormone replacement, *n* (%)	16 (5.5)	5 (6.1)	0.791
Aspirin, *n* (%)	12 (4.2)	1 (1.2)	0.313
Primary outcome			
Threatened abortion, *n* (%)	3 (1)	7 (8.5)	**0.001**

Data are presented as a number and percentage (%) of all cases or as median and interquartile range (IQR). *p* values have been calculated according to chi-square or Fisher exact test or Mann–Whitney test, when appropriate, and referred to as comparisons between study groups. Statistically significant correlations have been highlighted in bold character. Anti-Ac-terAA1 IgG: anti-c-terminus ApoA-1 autoantibody; APS: antiphospholipid syndrome; BMI: body mass index; CRP: C-reactive protein; GDM: gestational diabetes mellitus; PCOS: polycystic ovary syndrome; SLE: systemic lupus erythematosus.

**Table 4:** Linear regression analysis showing the association between anti-ApoA-1 IgG and anti-Ac-terAA1 IgG levels and threatened abortion.

	Univariate model			Multivariate model		
	*β*	95% CI	*p* value	*β*	95% CI	*p* value
Threatened abortion						
Anti-apoA-1 IgG	0.162	0.071-0.253	**0.001**	0.181	0.072-0.255	**<0.001**
Age				0.009	−0.003-0.004	0.854
Autoimmune diseases				−0.048	−0.096-0.035	0.354
Anti-Ac-terAA1 IgG	0.153	0.067-0.239	**0.001**	0.179	0.067-0.240	**0.001**
Age				−0.004	−0.004-0.003	0.943
Autoimmune diseases				−0.048	−0.096-0.035	0.355

Statistically significant *p* values are displayed in bold characters. Anti-ApoA-1 IgG: apoliprotein A-1 autoantibody; Anti-Ac-terAA1 IgG: anti-c-terminus apoA-1 autoantibody; CI: confidence interval.

## Data Availability

Due to ethical committee permission, we have to protect also anonymized data and ask for an Ethical Committee permission if database is required for checking.

## Supplementary Materials

Supplementary Materials Supplementary Table 1: maternal adverse outcomes other than threatened abortion of the overall cohort. Supplementary Table 2: maternal adverse outcomes other than threatened abortion according to positivity/negativity for ApoA-1 IgG autoantibodies. Supplementary Table 3: maternal adverse outcomes other than threatened abortion according to positivity/negativity for Ac-terAA1 IgG autoantibodies. Supplementary Figure 1: Spearman's rank correlation between anti-ApoA-1 and anti-Ac-terAA1 IgG levels.
