# Supplementary material for: Antiapolipoprotein A-1 Autoantibody Positivity Is Associated with Threatened Abortion
Source: Biomed Res Int. 2020 Mar 7;2020:9309121. doi: 10.1155/2020/9309121 (PMC7081016; doi:10.1155/2020/9309121)

Supplementary table 1. Maternal adverse outcomes other than threatened abortion of the overall cohort.

Pregnancy and delivery characteristics	
Gestational hypertension, n (%)	4 (1.1)
Gestational age at delivery, days	275 [270-282]
Premature delivery, n (%)	22 (6.6)
Cesarean delivery, n (%)	173 (50.7)
Urgent Cesarean delivery, n (%)	38 (11.1)
Overall maternal adverse outcomes, n (%)	184 (49.6)

Data are presented as a number and (%) of all cases or as median and [interquartile range].

Supplementary table 2. Maternal adverse outcomes other than threatened abortion according to positivity/negativity for ApoA-1 IgG autoantibodies.

	**Anti-ApoA-1 IgG negative (n=340)**	**Anti-ApoA-1 IgG positive (n=31)**	***p***
**Pregnancy and delivery characteristics**			
Gestational hypertension, n (%)	4 (1.2)	0	1.000
Gestational age at delivery, days, n (%)	275 [270-282]	275 [270-282]	0.956
Premature delivery, n (%)	22 (7.2)	0	0.237
Spontaneous delivery, n (%)	129 (41.2)	9 (32.1)	0.424
Induced delivery, n (%)	22 (7.0)	3 (10.7)	0.446
Cesarean delivery, n (%)	157 (50.2)	16 (57.1)	0.556
Urgent Cesarean delivery, n (%)	33 (10.5)	5 (17.9)	0.220
Overall maternal adverse outcomes, n (%)	167 (49.1)	17 (54.8)	0.578

Data are presented as a number and (%) of all cases or as median and [interquartile range].

p-values have been calculated according to Fisher exact test or Mann-Whitney *U* test, when appropriate, and referred to as comparison between study groups.

Anti-ApoA-1 IgG: Apoliprotein A-1 immunoglobulin G autoantibody.

Supplementary table 3. Maternal adverse outcomes other than threatened abortion according to positivity/negativity for Ac-terAA1 IgG autoantibodies.

	Ac-terAA1 IgG negative (n=289)	Ac-terAA1 IgG positive (n=82)	p
**Pregnancy and delivery characteristics**			
Gestational hypertension, n (%)	2 (0.7)	2 (2.4)	0.213
Gestational age at delivery, days	276 [270-283]	274 [269-282]	0.266
Premature delivery, n (%)	18 (6.9)	4 (5.6)	1.000
Spontaneous delivery, n (%)	103 (38.6)	35 (47.3)	0.183
Induced delivery, n (%)	19 (7.1)	6 (8.1)	0.802
Cesarian delivery, n (%)	141 (52.8)	32 (43.2)	0.151
Urgent Cesarian delivery, n (%)	33 (12.4)	5 (6.8)	0.214
Overall maternal adverse outcomes, n (%)	146 (50.5)	38 (46.3)	0.533

Data are presented as a number and (%) of all cases or as median and [interquartile range].

p-values have been calculated according to Fisher exact test or Mann-Whitney *U* test, when appropriate, and referred to as comparison between study groups.

Ac-terAA1 IgG: Ac-terAA1 IgG: anti-c-terminus ApoA-1 autoantibody.

Supplementary figure 1. Spearman's rank correlation between anti-ApoA-1 and anti-Ac-terAA1 IgG.

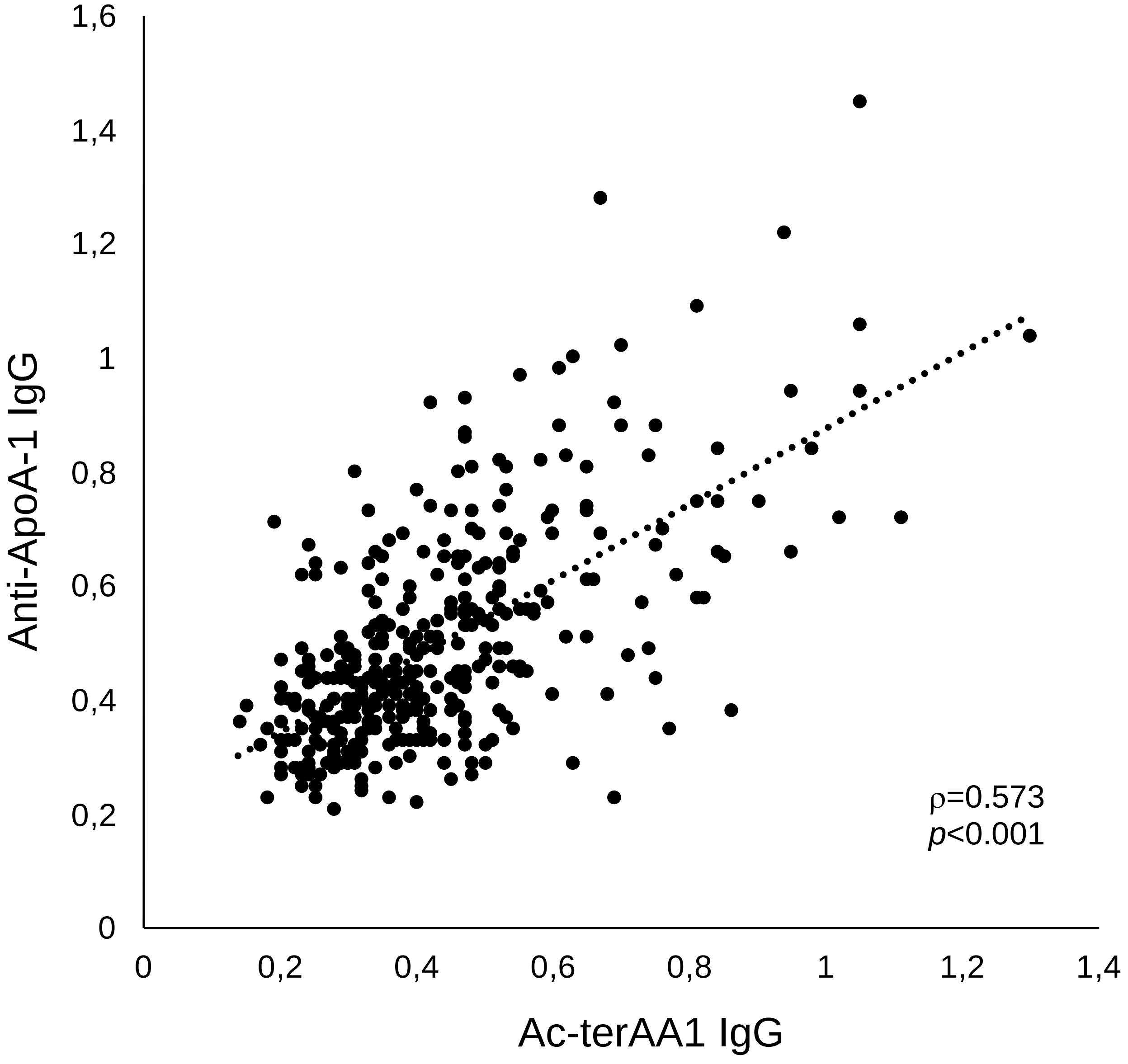

Supplement: Supplementary Materials — Supplementary Table 1: maternal adverse outcomes other than threatened abortion of the overall cohort. Supplementary Table 2: maternal adverse outcomes other than threatened abortion according to positivity/negativity for ApoA-1 IgG autoantibodies. Supplementary Table 3: maternal adverse outcomes other than threatened abortion according to positivity/negativity for Ac-terAA1 IgG autoantibodies. Supplementary Figure 1: Spearman's rank correlation between anti-ApoA-1 and anti-Ac-terAA1 IgG levels. [file 9309121.f1.pdf]
